# GO-mediated synergistic regulation of dispersion and acidity in WO_3_ for efficient long-chain α-olefin epoxidation

**DOI:** 10.1039/d6ra04930c

**Published:** 2026-07-06

**Authors:** Baoliang Lv, Wei Zhang, Guohua Zhang, Guofu Zhang, Miao Zhang, Shoufeng Xue, Huixiang Wang

**Affiliations:** a School of Chemistry and Chemical Engineering, Shanxi Normal University Taiyuan 030031 China lvbl@sxnu.edu.cn wanghx@sxnu.edu.cn; b Shanxi Key Laboratory of Coal-based Synthetic Oil Product Testing Changzhi 046000 China; c Shanxi Lu'an Coal-based Clean Energy Limited Liability Company Changzhi 046200 China

## Abstract

The epoxidation of long-chain α-olefins is an effective strategy for optimizing synthetic oil products and expanding their high-value-added derivatives. Tungsten trioxide (WO_3_) is a promising catalyst for this reaction, yet pure WO_3_ suffers from low specific surface area and a high density of Brønsted acid sites (B acid), resulting in poor activity and selectivity. Herein, graphene oxide (GO) was employed as a support to anchor WO_3_*via* an *in situ* growth strategy. Leveraging the strong interaction between the oxygen-containing functional groups of GO and the acidic hydroxyl groups of WO_3_, we achieved both high dispersion and effective reduction of B acid sites on WO_3_, successfully fabricating a high-performance WO_3_/GO catalyst. In the epoxidation of 1-octene, the WO_3_/GO catalyst exhibited excellent performance, specifically, its catalytic activity for producing 1,2-epoxyoctane increased from 7.9 mmol g_WO_3__^−1^ h^−1^ (pure WO_3_) to 20.3 mmol g_WO_3__^−1^ h^−1^, representing a 2.6-fold enhancement in catalytic efficiency. The catalyst also exhibited excellent performance towards other long-chain α-olefins. This study provides a simple, efficient, and dual-benefit strategy for the structural modification of WO_3_-based catalysts, offering a promising approach for designing low-acid, highly dispersed metal oxide catalysts for the efficient conversion of long-chain α-olefins.

## Abstract 2

*In situ* anchoring of WO_3_ onto GO enables high dispersion and reduced B acidity, boosting 1-octene epoxidation activity by 2.6 times.

## Graphical abstract

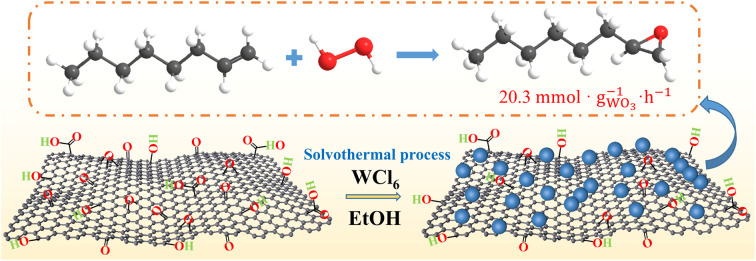

## 1. Introduction

Long-chain α-olefins (LAO) are key raw materials in the high-end chemical and materials industry, which cannot be produced on a large scale *via* traditional petrochemical routes.^1,2^ The Fe-based Fischer–Tropsch synthesis (Fe-FTS, synthetic oil) provides a viable alternative route for LAO production, with LAO (C_5+_) accounting for approximately 32% of the total products.^3^ However, due to the high separation cost of LAO-alkanes mixtures with similar carbon numbers, the Fe-FTS process directly hydrogenates the mixtures into oil, causing waste of these LAO resources.^4,5^ 1,2-Epoxyalkanes can serve as stabilizers and feedstocks for numerous high-quality fine chemicals, including epoxy resin, halogenated hydrocarbons, and detergents.^6,7^ Therefore, the epoxidation of LAO not only enhances the added value of Fe-FTS products, but also amplifies the differences in physical properties between alkenes and alkanes, potentially enabling efficient product separation and holding great significance for the synthetic oil industry.

With rising environmental consciousness, green olefin epoxidation has become increasingly necessary. Hydrogen peroxide (H_2_O_2_) is a desirable oxidant due to its benign byproduct (H_2_O) and its more facile activation compared with O_2_. Accordingly, the core of H_2_O_2_-based epoxidation lies in the developing efficient catalysts. Currently, homogeneous catalysts such as polyoxometalates ([XM_*x*_O_*y*_]^*n*−^, X = P, Si, As; M = W, Mo, *etc.*) and metal complexes (metal–porphyrin, metal–salan, methyltrioxorhenium, *etc.*) have been applied in the epoxidation of LAO (*e.g.*, 1-octene). Among these, the ammonium salt of [γ-SiW_10_O_36_]^8−^ exhibits the optimal catalytic activity, with both conversion and selectivity exceeding 90%.^8–12^ However, the practical application of these homogeneous catalysts is hindered by recovery difficult, high cost, and leaching of active components. Therefore, developing highly active heterogeneous catalysts for LAO epoxidation remains an urgent priority.

WO_3_ is an important solid acid material widely used in various catalytic reactions, including biomass conversion, hydrocarbon metathesis, and oxidation.^13–15^ It has also been successfully applied in the epoxidation of higher olefins using H_2_O_2_ as the oxidant. However, studies have primarily focused on highly reactive substrates such as cyclooctene, while investigations into the epoxidation of less reactive LAO remain limited.^16^ Therefore, developing efficient WO_3_-based catalysts for LAO epoxidation is highly desirable for both fundamental research and the synthetic oil industry.

For epoxidation reactions, the catalytic performance of bare WO_3_ is governed by two main factors: the number of active sites and the surface Brønsted acid (B acid) density. The former dictates the concentration of reactive intermediates, thereby determining olefin conversion; the latter, if excessive, can trigger ring-opening of the epoxide products, reducing selectivity.^17^ Based on these considerations, the preparation of high-performance WO_3_ catalysts requires increasing the number of active sites while reducing the B acid density. Increasing the specific surface area of WO_3_ can effectively enhance the number of active sites, and the solvothermal process is a favorable strategy to achieve this by suppressing rapid crystallization and growth, yielding WO_3_ with more defects, uniform size, and high surface area.^18–20^ Nevertheless, WO_3_ prepared *via* a solvothermal route still suffers from obvious agglomeration, which lowers its effective utilization.^21,22^ Combined with its relatively high B acid content, these factors jointly lead to unsatisfactory catalytic activity.^17^ Given the above facts, it would be lucky for the modification of WO_3_-based catalysts if there exists an approach that can simultaneously achieve a significant increase in the specific surface area of WO_3_ and a reduction in its B acid content.

GO has attracted significant attention in the field of energy conversion and storage due to its excellent high specific surface area and superior electron transport property.^23–25^ Moreover, owing to the abundance of oxygen-containing functional groups on its surface, GO also acts as an excellent functional support;^26,27^ therefore, *in situ* anchoring WO_3_ onto GO could be an effective strategy. Specifically, when GO is directly introduced into the solvothermal synthesis process of WO_3_, dehydration reactions will occur between its oxygen-containing functional groups and the hydroxyl groups (W–OH) of WO_3_ microcrystals. This leads to the anchoring and dispersed growth of WO_3_ on the GO surface, which can increase the specific surface area and improve the utilization efficiency of WO_3_. Estananto *et al.*^28^ have introduced GO during the solvothermal process to support WO_3_, but the excessive WO_3_ loading resulted in poor dispersion in the as-obtained WO_3_/GO sample. In addition, the B acid sites of WO_3_ mainly originate from its surface W–OH, which will be effectively consumed during the *in situ* anchoring process, thereby reducing the B acidity of WO_3_. Therefore, the anchoring WO_3_ on GO is theoretically expected to simultaneously increase specific surface area of WO_3_ and decrease its B acid density, making the WO_3_/GO a promising catalyst for LAO epoxidation.

In this work, electrochemically expanded GO with a moderate specific surface area was used as a support, ethanol as solvent, and a WO_3_/GO catalyst with a loading of 15 wt% was prepared *via* a solvothermal process based on our previous study.^29^ The structures of GO, WO_3_ and WO_3_/GO were characterized by various techniques. The 1-octene epoxidation was used as a model reaction, and a preliminary structure–activity relationship of the catalyst was established in combination with the catalytic performance.

## 2. Experimental section

### 2.1. Materials

All experimental chemical reagents were of analytical grade and used without further purification. Tungsten chloride (WCl_6_, 99%), 1-octene (C_8_H_16_, 98%) and anisole (C_7_H_8_O, 99%) were purchased from Shanghai Aladdin Biochemical Technology Co., Ltd. Anhydrous ethanol (EtOH, 99%), acetonitrile (CH_3_CN, 99%) and hydrogen peroxide (H_2_O_2_, 30 wt%) were purchased from Sinopharm Group Chemical Reagent Co., Ltd. GO slurry (3.7 mg g^−1^) was purchased from Yanpai (Shanxi) Technology Service Co., Ltd. Distilled water with a resistance of 18.2 MΩ cm^−1^ was used throughout the experiments.

### 2.2. Purification and powder conversion of GO slurry

200.0 g of GO slurry was poured into a beaker and rinsed with ultrapure water until completely neutral. The resulting thick slurry was then transferred to an evaporating dish, sealed with plastic wrap, pierced with small holes, and placed in a freezer overnight to freeze. After freeze-drying for 12 h, it was kept at 30 °C for 6 h to obtain GO powder, which was stored in a sample tube for further use.

### 2.3. Preparation of pure WO_3_

0.8 g of WCl_6_ was dissolved in 50 mL ethanol to form a yellow transparent solution, then transferred into a 100 mL stainless steel autoclave, sealed, fixed in a homogeneous reactor, and maintained at 160 °C under rotation (25 r min^−1^) for 10 h. After naturally cooling to room temperature, the solid product was collected by filtration and washed alternately with deionized water and ethanol three times, and dried at 60 °C overnight to obtain the solvothermally prepared pure WO_3_.

### 2.4. *In situ* anchoring and growth of WO_3_ on GO surface

0.3 g of GO was dispersed in 30 mL of ethanol under stirring, followed by dropwise addition of a yellow solution containing 77.0 mg WCl_6_ in 20 mL ethanol. After continuous stirring for 3 h, the mixture was transferred into a 100 mL stainless steel autoclave, sealed, fixed in a homogeneous reactor, and maintained at 160 °C under rotation (25 r min^−1^) for 10 h. After naturally cooling to room temperature, the solid product was collected by filtration, washed alternately with deionized water and ethanol three times, and freeze-dried for 12 h to obtain the WO_3_/GO.

### 2.5. Physical characterizations

X-ray powder diffraction (XRD, SmartLab diffractometer) was performed using monochromatic Cu Kα radiation (*λ* = 1.54056 Å, 40 kV, 40 mA) at a scanning speed of 10° min^−1^ in the 2*θ* range of 10–90° to detect the crystal structures of products. The morphology, size, and surface element distribution of samples were observed using scanning electron microscopy and transmission electron microscopy (SEM, JSM-IT800 (SHL); TEM, FEI Talos F200X G2) and an energy dispersive spectrometer (EDS), and the sample was fixed on conductive Cu tape in the SEM. The specific surface area was measured using a physical adsorption analyzer (BET, Micromeritics3Flex). Surface elemental composition and valence state were determined using X-ray photoelectron spectroscopy (XPS, Thermo Scientific K-alpha+) at room temperature under α-radiation. Raman spectra were carried out (Raman, InVia) using a laser wavelength of 514.5 nm and Fourier transform infrared spectroscopy (FT-IR, Nicolet IS50) was performed on a MCT detector and collected over the range of 400−4000 cm^−1^. The acid strength of the samples was measured by ammonia temperature-programmed desorption (NH_3_-TPD, 7.5% NH_3_/He, Microtrac BEL) and the types of acid sites were characterized by *in situ* ammonia infrared spectroscopy (*In situ* NH_3_-FTIR, 1.0% NH_3_/He, FTIR-850) at 70 °C.

### 2.6. Catalytic activity testing

The epoxidation reaction of 1-octene was carried out as follows: 4.0 mmol 1-octene, 4.0 mmol H_2_O_2_ (30 wt%), 50 mg of catalyst, and 10 mL of acetonitrile were added into a 25 mL reaction autoclave liner. The mixture was ultrasonicated for 3 min, then sealed into the stainless steel autoclave shell. The autoclave was fixed in a homogeneous reactor and stirred at 50 r min^−1^ and 70 °C for 8 h. After the reaction, 0.2 g of anisole was added as an internal standard. The mixture was stirred, centrifuged, and the supernatant was analyzed by gas chromatography (GC-920) to determine the conversion of 1-octene and the selectivity toward products. During the experiment, qualitative analysis was performed using gas chromatography–mass spectrometer (GC–MS QP-2050).

## 3. Results and discussion

### 3.1. Microstructure analysis

**Fig. 1:**
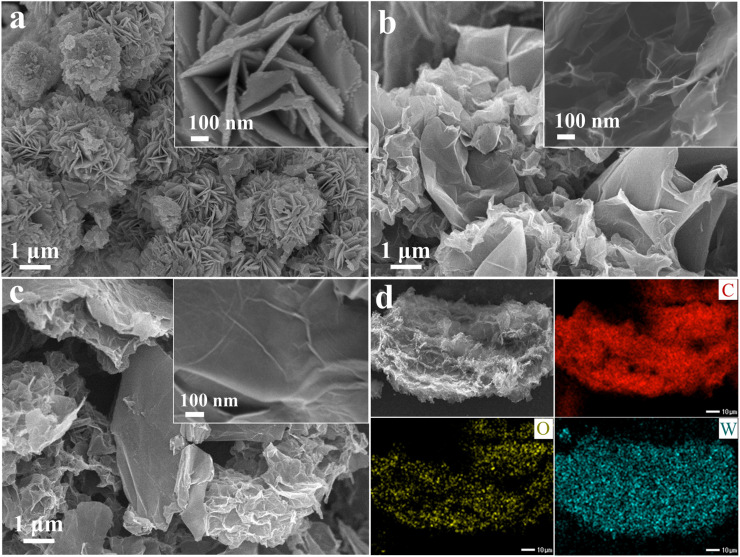
The SEM images of (a) WO_3_, (b) GO, (c) WO_3_/GO samples and (d) EDS-mapping of WO_3_/GO composite.

The SEM images of pure WO_3_, pristine GO and WO_3_/GO composite are shown in Fig. 1. As seen in Fig. 1a that WO_3_ exhibits a micron-sized flower-like structure self-assembled from numerous nanosheets, showing good uniformity but severe agglomeration. A higher-magnification view (inset of Fig. 1a) reveals that the nanosheets have a thickness of 20–70 nm and a lateral size of hundreds of nanometers, with rough edges and numerous steps. Fig. 1b shows that pristine GO presents a typical two-dimensional wrinkled lamellar structure with a sponge-like morphology, which provides sufficient specific surface area for the dispersion of active components. According to Fig. 1c, the lamellar structure of GO remains intact after WO_3_ loading, yet its sheets tend to overlap. This phenomenon probably arises from interlayer interactions of GO and interfacial interactions between GO and WO_3_ during solvothermal treatment, which may lead to a reduction in the specific surface area of GO. Furthermore, no distinct WO_3_ particles are visible in the SEM image, indicating their extremely small sizes.

To verify the dispersion state of WO_3_ on the GO surface, elemental mapping analysis (SEM-EDS-mapping, Fig. 1d) was performed on the WO_3_/GO sample. For better visualization of the C element distribution, conductive Cu tape was used as the substrate. The results show that C, O and W are uniformly distributed throughout the composite, with the distribution of W perfectly matching the contours of the GO sheets, showing no sign of local aggregation.

**Fig. 2:**
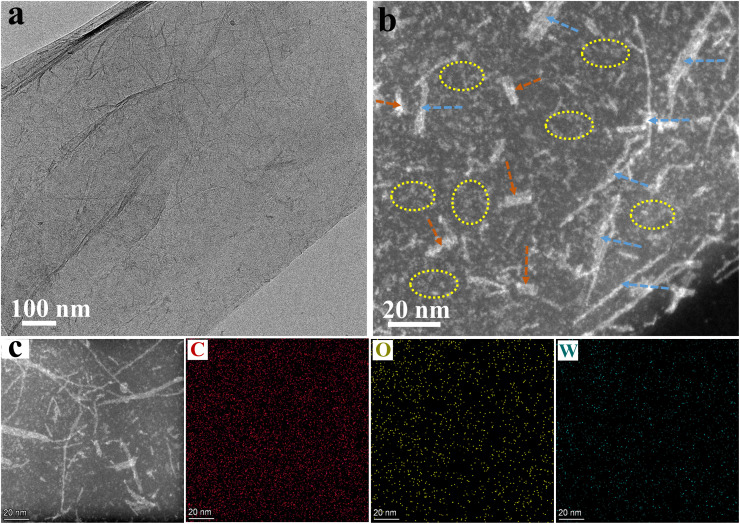
(a and b) TEM images of WO_3_/GO sample and (c) corresponding EDS mapping.

The microstructure of WO_3_ particles can be observed by TEM, as shown in Fig. 2a and b. The images reveal that WO_3_ is highly dispersed on the GO surface in the form of extremely small nanoparticles (the ultrafine particles within the yellow dashed circles) and nanowires of varying lengths (indicated by blue and orange arrows), which are composed of these nanoparticles. The EDS-mapping images show that W and O elements are very uniformly dispersed on the GO surface, further demonstrating the homogeneous distribution of WO_3_ and confirming that the introduction of GO support indeed greatly improves the dispersion of the active component, thereby providing more active sites for catalysis. In addition, the mass ratios of C, O, and W elements are 68.6%, 23.5%, and 8.0%, respectively, in agreement with the designed loading, and the excess oxygen originates from the remaining oxygen-containing functional groups on GO. These results indicate that “high dispersion”, one of the intended goals of the experimental design, has been successfully achieved.

**Fig. 3:**
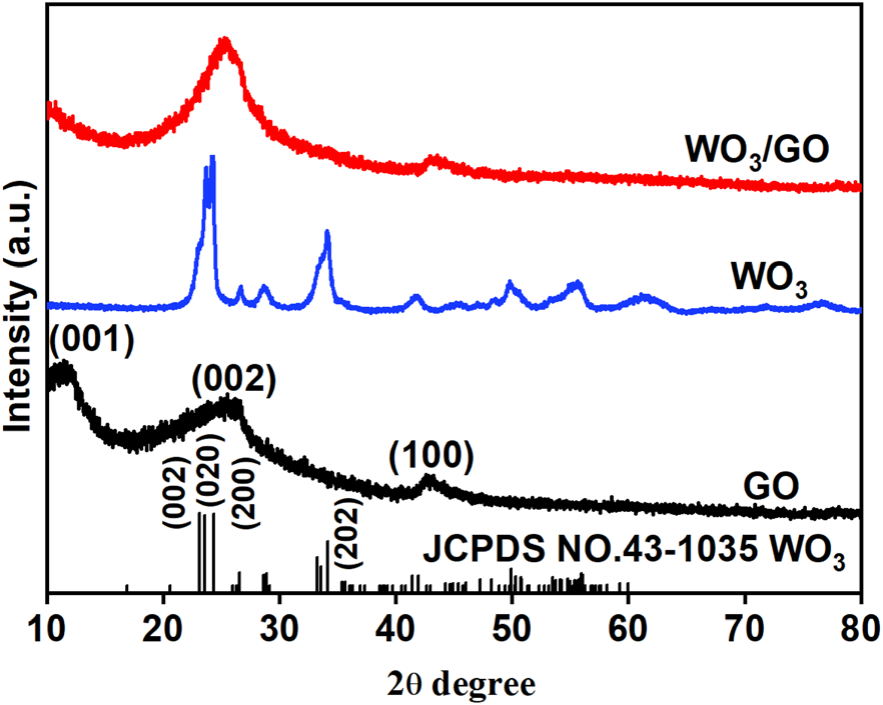
XRD patterns of GO, WO_3_ and WO_3_/GO samples.

The XRD patterns of three samples were displayed in Fig. 3. The diffraction peaks of GO at 11.6°, 25.4° and 42.8° are assigned to the (001), (002), and (100) planes, respectively. For pure WO_3_, the characteristic peaks located at 23.1°, 23.5°, 24.4°, and 34.2° correspond to the (002), (020) (200) and (202) planes of monoclinic WO_3_ (JCPDS No. 43-1035), respectively. For the WO_3_/GO sample, the (001) diffraction peak of GO, arising from the interlayer spacing expansion due to oxygen-containing functional groups, disappears after WO_3_ growth.^30^ This can be ascribed to the consumption of these functional groups *via* their interaction with WO_3_, which disrupts the ordering of GO (001) plane and thus eliminates its diffraction peak. In addition, no obvious diffraction peaks of WO_3_ are detected, which may be attributed to the formation of WO_3_ with low crystallinity and small particle size. Meanwhile, the (002) diffraction intensity of GO increases to some extent, suggesting a contribution to the diffraction intensity of WO_3_.

**Fig. 4:**
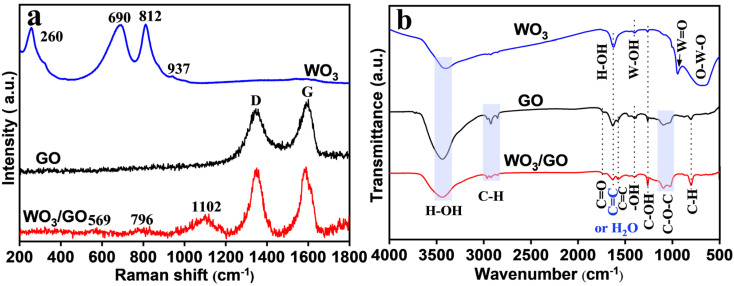
The (a) Raman and (b) FT-IR spectra of pure WO_3_, GO and WO_3_/GO samples.

Raman spectroscopy is an effective technique for detecting chemical bond changes in metal oxides and disorder in carbon-based materials *via* its G and D band features.^31,32^ Accordingly, the Raman analysis of the samples was conducted, and the results are presented in Fig. 4a. For pure WO_3_, the peak at 260 cm^−1^ is assigned to the O–W–O bending vibration of bridging oxygen, while the peaks at 690 cm^−1^ and 812 cm^−1^ correspond to O–W–O stretching vibrations. Additionally, the peak at 937 cm^−1^ is attributed to the terminal W=O stretching vibration.^33^ In WO_3_/GO, two weak O–W–O stretching vibration peaks shift to lower wavenumbers of 569 cm^−1^ and 796 cm^−1^; the W=O peak at 937 cm^−1^ disappears, and a strong peak emerges at 1102 cm^−1^, which is most likely be assigned to the vibration of a newly formed C–O–W bond.^34,35^ GO exhibits characteristic D and G peaks at 1345 cm^−1^ and 1595 cm^−1^, with an *I*_D_/*I*_G_ ratio of 0.9, which increases to 1.04 for WO_3_/GO, indicating increased defects after loading WO_3_. This structural change is proposed to arise from chemical bonding between WO_3_ nanoparticles and GO functional groups, which disrupts the sp^2^ conjugated structure of the graphene framework and creates new defect sites, leading to the enhanced D peak.^36^

FT-IR spectrum was also used to detect and analyze the chemical bonds in the samples, as shown in Fig. 4b. For pure WO_3_, the bands at 3408 and 1618 cm^−1^ are assigned to stretching and bending vibrations of –OH groups from adsorbed water. The peaks at 1397, 1253, 945, and 667 cm^−1^ correspond to stretching vibrations of W–OH, C–OH (possibly originating from surface organic contamination), W=O, and O–W–O.^37^ Pristine GO exhibits bands at 3440, 2963 (shaded), 1735, 1630, 1578, 1397, 1261, 1060 (shaded), and 800 cm^−1^, assigned to the stretching vibrations of –OH, C–H, C=O, C=C, C–OH, C–O–C, and =C–H.^26,38^ In the WO_3_/GO sample, the intense band at 1397 cm^−1^ reveals that C–OH groups are retained during the solvothermal process. Furthermore, enhanced band at 800 cm^−1^ suggests the presence of an additional O–W–O stretching vibration, which possibly overlaps with C–O–W vibration.

**Fig. 5:**
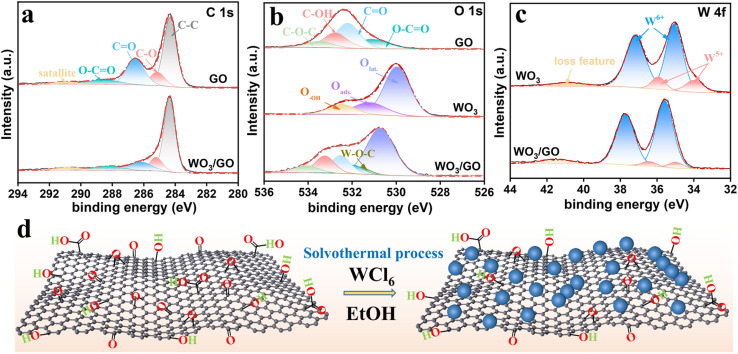
XPS spectra of samples: (a) C 1s, (b) O 1s, (c) W 4f; (d) the schematic diagram of GO and WO_3_ growth anchoring process.

XPS spectrum can identify variations in surface functional groups, elemental contents, and chemical valence states of the samples. Fig. 5a shows the C 1s spectra of GO, where peaks at 284.3, 285.1, 286.5, 288.5, and 290.9 eV correspond to C–C, C–O, C=O, O–C=O, and the C 1s satellite peak.^39^ After loading WO_3_ on GO surface, the C=O content decreases significantly. This phenomenon is also reflected in the O 1s spectra of GO and WO_3_/GO (Fig. 5b). The C–OH content shows no obvious decrease after WO_3_ loading, which is consistent with the FT-IR findings. For pure WO_3_, peaks at 530.0, 531.2, and 532.4 eV are ascribed to lattice oxygen (O_–latt._), adsorbed oxygen associated with oxygen vacancies (O_–ads._), and oxygen from adsorbed water (O_–OH_), respectively. However, the peaks for O_–ads._ and O_–OH_ are difficult to deconvolute in the WO_3_/GO sample;^40^ additionally, the peak at 531.6 eV is most likely attributed to the binding energy of C–O–W, a finding supported by similar result reported by Sivkov.^41^ The abundance of oxygen vacancies on WO_3_ surface can be reflected by the W^5+^content, which is quantified as the peak area ratio of W^5+^ to the total of W^5+^ and W^6+^ (W^5+^/(W^5+^ + W^6+^)). From the W 4f spectra in Fig. 5c, it can be calculated that the proportion of W^5+^ decreases from 14.76% in pure WO_3_ to 6.28% in the WO_3_/GO after GO introduction, indicating that oxygen derived from the oxygen-containing functional groups of GO fills some oxygen vacancies in WO_3_ during the loading process. Furthermore, the binding energies of both W and O shift toward higher values after hybridization, suggesting an overall decrease in the electron cloud density of WO_3_, and such variation is conducive to the adsorption and activation of the oxidant H_2_O_2_. Based on the above results, the WO_3_ microcrystals can readily anchor and grow on GO surface through dehydration reaction between acidic W–OH and oxygen-containing functional groups, accompanied by the formation of C–O–W bonds, and the corresponding loading process is schematically illustrated in Fig. 5d.

**Fig. 6:**
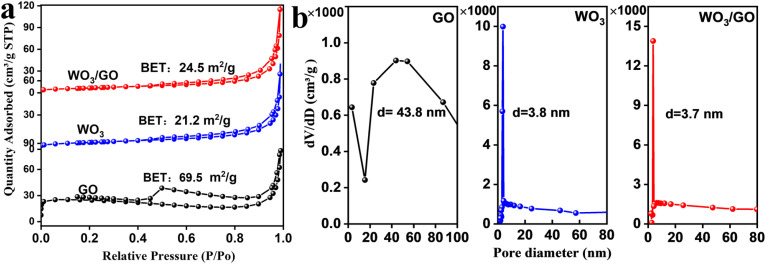
(a) N_2_ adsorption–desorption isotherms and (b) pore distributions of GO, WO_3_, and WO_3_/GO catalysts.

Fig. 6a displays the N_2_ adsorption–desorption isotherms of the three materials. All curves belong to type IV with distinct H_3_ hysteresis loops at medium-to-high relative pressure region (*P*/*P*o > 0.4), demonstrating coexisting meso- and macropores derived from slit-shaped pores formed by particle or sheet stacking. The BET surface areas of GO, WO_3_ and WO_3_/GO are 69.5, 21.2 and 24.5 m^2^ g^−1^, respectively. GO exhibits the most pronounced hysteresis loop, consistent with its abundant interlayer voids and large surface area. WO_3_/GO exhibits a much weaker hysteresis loop. This is mainly attributed to the overlapping of GO sheets during the loading process, which ultimately reduces the specific surface area of the sample to 24.5 m^2^ g^−1^. Notably, the dense distribution of WO_3_ nanoparticles on GO contributes substantially to the surface area of the composite. Meanwhile, pure WO_3_ exhibits the lowest specific surface area of 21.2 m^2^ g^−1^. The pore size distributions are presented in Fig. 6b, revealing that GO has a broad pore-size distribution with a main peak at 43.8 nm, corresponding to the large slit pores from GO sheet stacking; pure WO_3_ has a uniform nanoflower hierarchical structure, giving a narrow pore size centered at 3.8 nm; WO_3_/GO exhibits a very narrow pore size distribution centered at 3.7 nm (typical mesopores), indicating reduced interlayer spacing of GO and uniform WO_3_ nanoparticles. Overall, the WO_3_/GO composite achieves high dispersion of WO_3_ and the retained mesoporous structure will facilitate the diffusion of reactants and products, thereby enhancing catalytic performance.

**Fig. 7:**
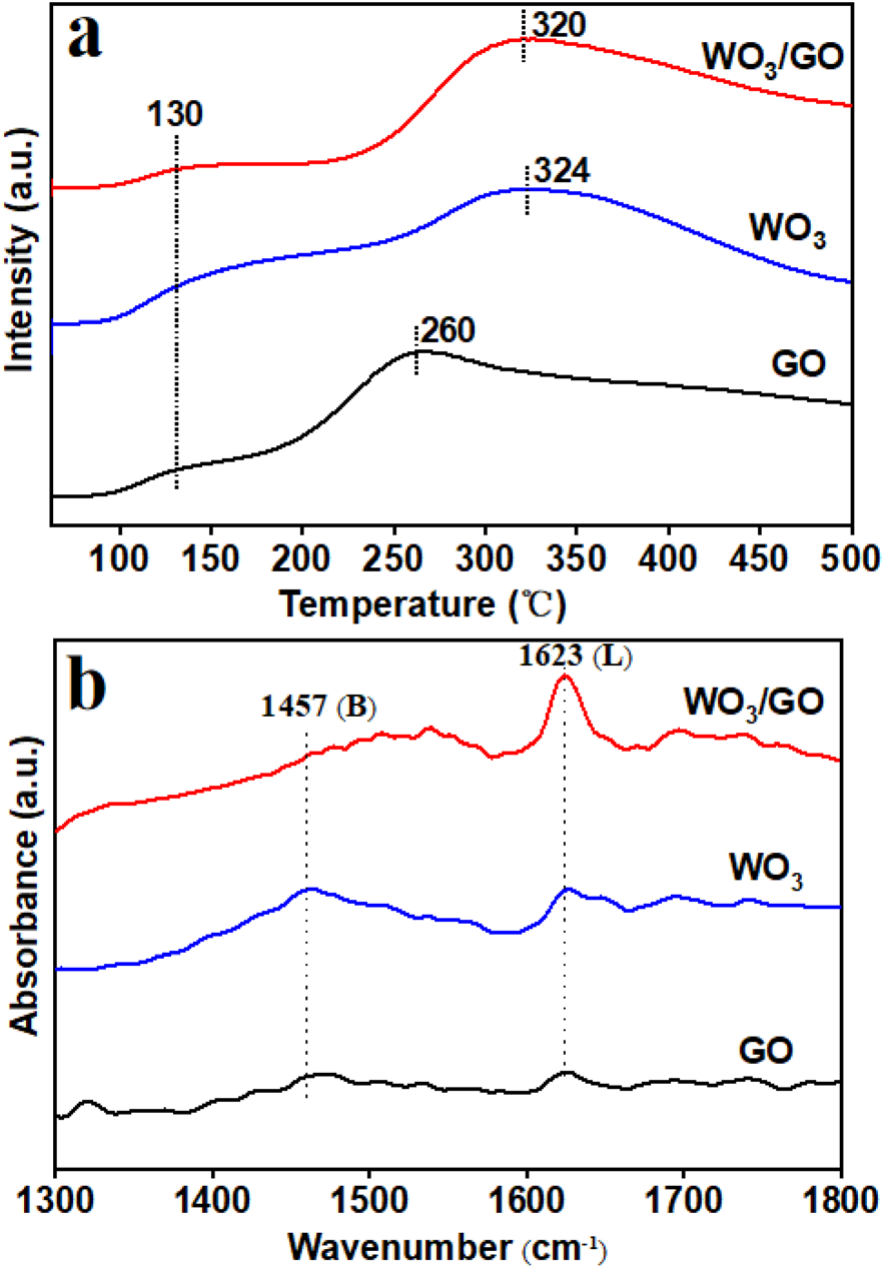
(a) NH_3_-TPD and (b) *in situ* NH_3_-FTIR spectra of the catalysts.

Subsequently, the acidic properties of as-prepared catalysts were characterized by NH_3_-TPD, and the results are presented in Fig. 7a. All three samples display two NH_3_ desorption peaks: the first peak, appearing at 100–200 °C, is assigned to weak acid derived from B acid sites, while the second peak, in the range of 200–450 °C, corresponds to medium-strong acid originating from Lewis acid sites.^42^ Notably, GO itself displays a distinct desorption feature of medium-strong acid, which is primarily attributed to its large specific surface area, as a large amount of physically adsorbed NH_3_ may form stable salts with surface carboxylic acid groups at high temperatures. In addition, the edge carbon sites may also exert strong coordination interactions with NH_3_.^43^ Pure WO_3_ contains both weak B acid sites and more abundant medium-strong L acid sites. Upon loading WO_3_ onto GO, the B acid content of WO_3_ decreases markedly, whereas the variation in L acid content is obscured by signal interference from GO.

*In situ* NH_3_-FTIR is also an effective technique for probing changes in different acid sites on solid catalysts. After NH_3_ adsorption on the catalysts, the absorption peaks at 1623 cm^−1^ and 1457 cm^−1^ are assigned to L acid and B acid sites on catalyst surface, respectively.^44^ As shown in Fig. 7b, GO exhibits only very weak peaks at these two wavenumbers, which is inconsistent with the NH_3_-TPD results. This discrepancy is primarily attributable to differences in test methods: *In situ* NH_3_-FTIR characterization employs low-concentration NH_3_ and vacuum treatment after adsorption to eliminate most physically adsorbed NH_3_ and retain only chemically adsorbed species. Compared with pure WO_3_, WO_3_/GO exhibits an enhanced L acid signal but a diminished B acid signal, indicating that the loading process effectively reduces the content of B acid. Thus, the second purpose of the catalyst design—reducing B acid—has been achieved.

### 3.2. Catalytic epoxidation performance

Under the preliminary experimental conditions (70 °C, 8 h), the catalytic performances of WO_3_ catalysts before and after loading were evaluated in the epoxidation of 1-octene, and the results are shown in Table 1. When the catalyst amount was 40.0 mg, pure GO showed no activity under the same conditions, and the conversion of 1-octene and selectivity toward 1,2-epoxyoctane for pure WO_3_ are 49.8% and 53.3%, respectively, giving a yield of 26.5% and a 1,2-epoxyoctane productivity (*P*_Epo_) of 3.3 mmol g_WO_3__^−1^·h^−1^; this low selectivity is mainly attributed to the abundant B acid sites on the surface of pure WO_3_. In contrast, the WO_3_/GO exhibits good performance, achieving a conversion of 26.7%, selectivity of 94.5%, yield of 25.3% and *P*_Epo_ of 18.9 mmol g_WO_3__^−1^·h^−1^. Furthermore, as shown in the table, the conversion rises with increasing catalyst dosage. Nevertheless, both the conversion of 1-octene and selectivity toward 1,2-epoxyoctane decrease when the catalyst dosage reaches 60.0 mg: an excessive catalyst dosage hinders mass transfer of reactants and products, thereby reducing conversion; meanwhile, the concentrated and unconsumed B acid sites promote ring-opening side reactions and overoxidation, thus lowering selectivity. Hence, 50.0 mg is identified as the optimal dosage for this reaction system, affording a conversion of 32.0%, selectivity of 94.9%, yield of 30.4% and *P*_Epo_ of 20.3 mmol g_WO_3__^−1^·h^−1^.

**Table 1:** Epoxidation performance of WO_3_ and WO_3_/GO catalysts toward 1-octene^a^

Entry	Catalyst	Dosage (mg)	Conv. (%)	Select. (%)				Yield (%)	*P*_Epo_ (mmol g_WO_3__^−1^·h^−1^)
				Epoxide	Glycol	Acid	Others		
1	WO_3_	40	49.8	53.3	11.9	22.1	12.7	26.5	3.3
2	WO_3_/GO	40	26.7	94.5	0	0	5.5	25.3	18.9
3	WO_3_/GO	30	16.3	94.8	0	0	5.2	15.5	10.4
4	WO_3_/GO	50	32.0	94.9	0	0	5.1	30.4	20.3
5	WO_3_/GO	60	30.5	90.1	0	3.2	6.7	27.5	18.3
6	WO_3_	7.5	16.4	72.2	9.3	12.7	5.8	11.8	7.9

a Reaction conditions: 4 mmol of 1-octene, 4 mmol of H_2_O_2_ (30 wt%), 10 mL of CH_3_CN, 70 °C, 8 h. Epoxide productivity (*P*_Epo_) = *n*(epoxide)/(*m*(WO_3_)·*t*(reaction time)), mmol g_WO_3__^−1^·h^−1^.

For 50.0 mg of the WO_3_/GO composite catalyst, the loaded WO_3_ mass is approximately 7.5 mg. Under this loading, pure WO_3_ exhibits a conversion of 16.4% and selectivity of 72.2%, corresponding to a yield of 11.8% and *P*_Epo_ of 7.9 mmol g_WO_3__^−1^·h^−1^. This indicates that with a small amount of catalyst, 1,2-epoxyoctane is less likely to contact the B acid sites, thereby effectively suppressing side reactions. However, the limited number of active sites results in lower conversion. These results clearly demonstrate that immobilizing WO_3_ on the GO surface not only improves WO_3_ dispersion but also reduces its B acid content, thereby enhancing catalytic performance and significantly improving the utilization efficiency of WO_3_.

Substrate scope experiments were subsequently conducted, and the results are given in Table 2. The WO_3_/GO catalyst also exhibits catalytic activity toward other long-chain α-olefins, including 1-hexene, 1-decene and 1-dodecene, with corresponding conversions of 38.2%, 20.1%, and 14.7%, respectively, all achieving satisfactory selectivities above 93%. Additionally, it shows better catalytic performance for cyclohexene and cyclooctene, with conversions of 40.6% and 56.7%. Cyclohexene shows a relatively low selectivity of 65.1%, while cyclooctene achieves a selectivity of 100%. Notably, the catalyst displays higher activity toward cyclooctene than cyclohexene, which is mainly attributed to the greater stability of the six-membered ring which hinders the epoxidation reaction.

**Table 2:** Epoxidation performance of WO_3_/GO catalyst toward different olefins^a^

Entry	Substrate	Product	Conv. (%)	Select. (%)		Yield (%)	*P*_Epo_ (mmol g_WO_3__^−1^·h^−1^)
				Epoxide	Others		
1	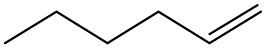	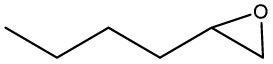	38.2	96.9	3.1	37.0	24.7
2	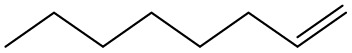	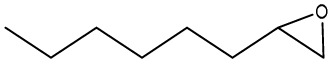	32.0	94.9	5.1	30.4	20.3
3	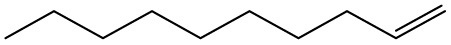	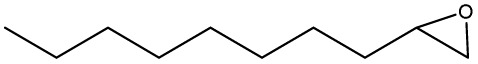	20.1	93.6	7.4	18.8	12.5
4	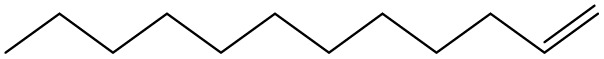	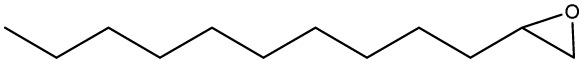	14.7	94.2	5.8	13.8	9.2
5	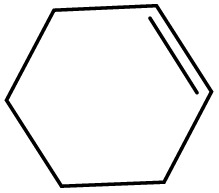	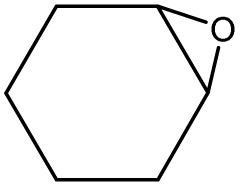	40.6	65.1	34.9	26.4	17.6
6	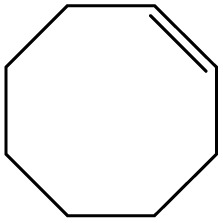	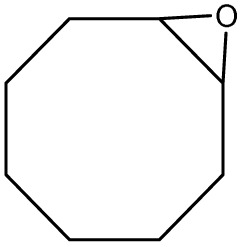	56.7	100	0	56.7	37.8

a Reaction conditions: 50 mg of catalyst, 4 mmol olefin 4 mmol of H_2_O_2_ (30 wt%), 10 mL of CH_3_CN, 70 °C, 8 h. These olefins were purchased from Shanghai Aladdin Biochemical Technology Co., Ltd.

**Fig. 8:**
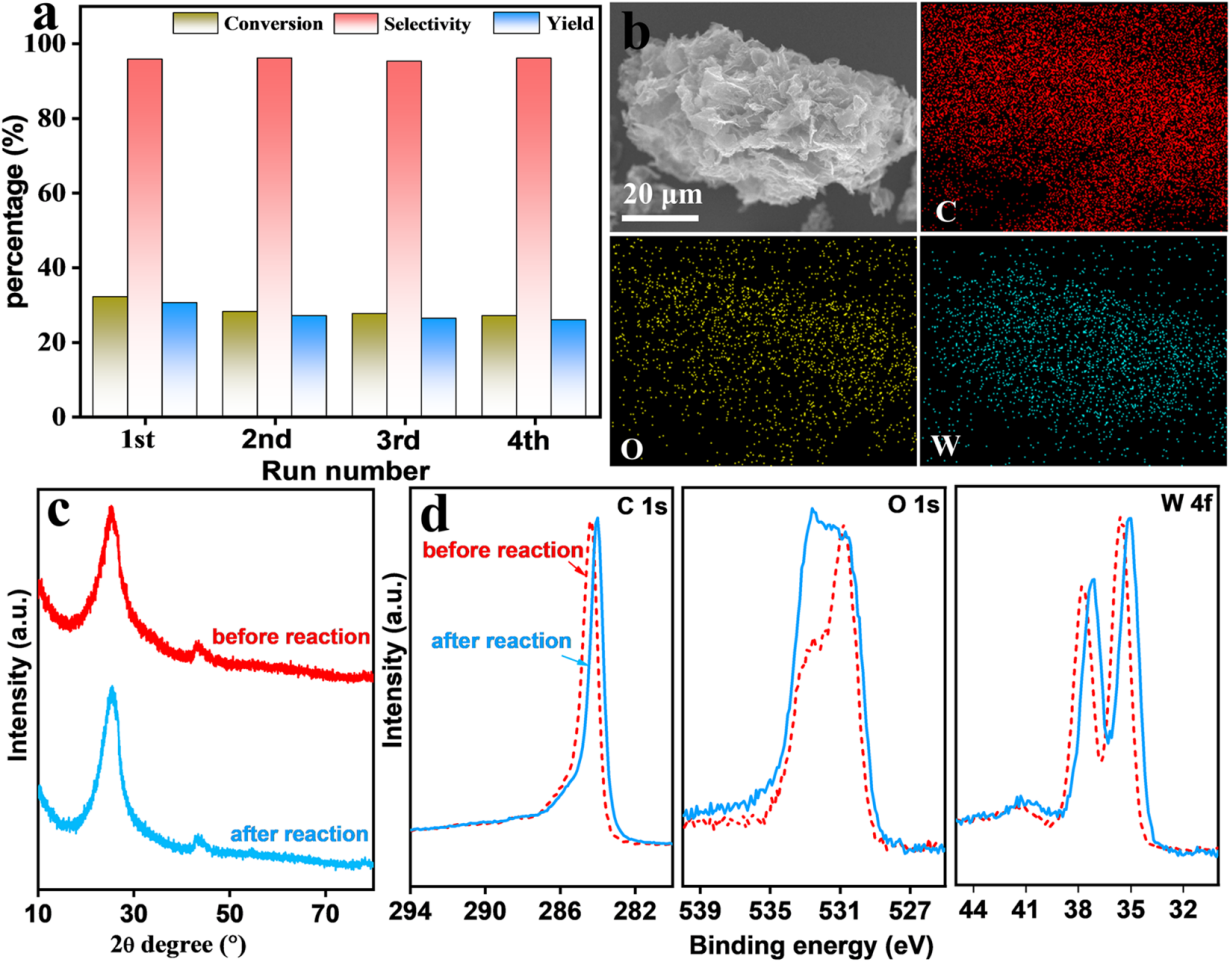
(a) Cyclic stability test of WO_3_/GO catalyst; (b) SEM image with EDS–mapping, (c) XRD patterns and (d) XPS (C 1s, O 1s and W 4f) spectra of fresh WO_3_/GO and used WO_3_/GO.

The recycling stability of the WO_3_/GO catalyst was evaluated over four consecutive cyclic reactions (Fig. 8a). Notably, the conversion shows a slight drop in the second cycle, likely due to the deactivation of partial unstable active sites during the first reaction. Subsequently, both conversion and product yield remain basically stable throughout the subsequent cycles, while the selectivity maintains nearly constant in the whole recycling process. The structural properties of the recycled catalyst were further characterized, as displayed in Fig. 8b–d: WO_3_ nanoparticles remain uniformly dispersed on the GO surface (Fig. 8b), and no significant changes are observed in the XRD diffraction peaks (Fig. 8c), demonstrating that the bulk structure and crystal phase of the catalyst are well preserved after cyclic reactions; XPS analysis (Fig. 8d) reveals only negligible shifts in the binding energies of C 1s and W 4f. However, the signal intensity in the range of 531–535 eV in the O 1s spectrum increases remarkably, which is assigned to the residual oxygen-containing products strongly adsorbed on the catalyst surface that have not been completely removed after reaction. Furthermore, electron-donating groups such as methyl and methylene in the surface-adsorbed organic substances could transfer electrons to the catalyst surface, leading to a slight shift in binding energies. Collectively, the as-prepared WO_3_/GO catalyst possesses excellent structural and surface chemical stability during the catalytic reaction, thus endowing it with excellent recyclability.

### 3.3. Catalytic epoxidation mechanism

Above analysis reveals that anchoring WO_3_ onto GO diminishes its surface B acidity, predominantly at the interface. Benefiting from the inherent wrinkled structure of GO nanosheets, the contact area between WO_3_ and GO is substantially increased, which further facilitates adequate consumption and passivation of interfacial W–OH sites (B acid sites). This process reconstructs the acid site distribution on WO_3_: at the interfacial side, massive B acid sites are eliminated, while Lewis acid sites (such as W^5+^ species and surface oxygen vacancies) are largely retained; on the non-interfacial side, a portion of pristine W–OH sites are preserved on the WO_3_ surface, but because of the dense nanoparticle packing, many of the unconsumed W–OH sites located between the particles may be stabilized *via* forming hydrogen bonds with one another. Such an asymmetric distribution of acid sites provides a structural foundation for the excellent catalytic selectivity in the subsequent epoxidation reaction.

**Fig. 9:**
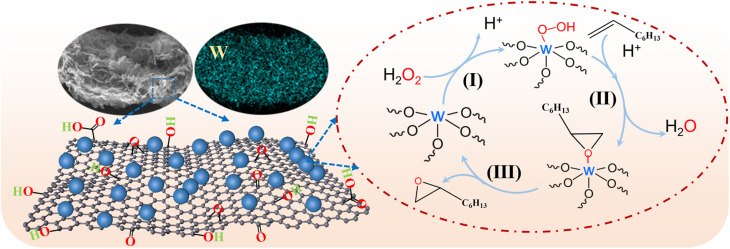
The proposed epoxidation mechanism of WO_3_/GO catalyst.

Previous reports and our recent studies have demonstrated that W–OOH serves as the key active intermediate for olefin epoxidation over WO_3_-based catalysts, which is primarily generated at oxygen vacancy sites.^45,46^ Accordingly, a proposed epoxidation mechanism of WO_3_/GO is illustrated in Fig. 9. Specifically, H_2_O_2_ is adsorbed and activated at oxygen vacancies to form reactive W–OOH species (I). Subsequently, the electrophilic oxygen attacks the C=C double bond of olefin and accomplishes oxygen insertion, generating adsorbed epoxide intermediates (II). Finally, the target product desorbs, and the active sites of the catalyst are regenerated for continuous catalysis.

Notably, although partial oxygen vacancies are filled or passivated by the strong interfacial interaction after WO_3_ immobilization on GO, WO_3_ exists as ultrafine nanoparticles with extremely high dispersion on the GO substrate. Consequently, the number of accessible oxygen vacancies per unit mass of WO_3_ catalyst does not decrease significantly. Instead, the improved dispersion endows the composite with enhanced conversion. Furthermore, since the anchoring process consumes a large amount of B acid sites, the hydrolysis of epoxide is suppressed, thereby increasing the product selectivity.

## 4. Conclusion

Although WO_3_ is a promising catalyst for the epoxidation of long-chain α-olefins to produce high-value synthetic oil derivatives, its practical application is severely constrained by low specific surface area and an overabundance of B acid sites. In this work, graphene oxide (GO) was directly introduced as a support during the solvothermal growth of WO_3_. Owing to the strong interaction between oxygen-containing functional groups on GO and acidic hydroxyl groups of WO_3_, highly dispersed immobilization of WO_3_ was achieved, while the B acid content of WO_3_ was effectively reduced. As a result, the as-fabricated WO_3_/GO composite exhibited remarkably enhanced catalytic activity toward the epoxidation of 1-octene. Specifically, the conversion of 1-octene and selectivity toward 1,2-epoxyoctane were 32.0%, 94.9%, respectively, and the formation capacity of 1,2-epoxyoctane increased from 7.9 mmol g_WO_3__^−1^·h^−1^ for pure WO_3_ to 20.3 mmol g_WO_3__^−1^·h^−1^, greatly improving the utilization efficiency of WO_3_. Moreover, the WO_3_/GO catalyst delivered high selectivity toward other long-chain olefins and possessed favorable structural stability. This study provides a simple, dual-purpose strategy for the performance optimization and modification of WO_3_-based catalysts.

## Author contributions

Baoliang Lv: writing–original draft, writing–review & editing, formal analysis, conceptualization, funding acquisition. Wei Zhang: investigation, writing–original draft, formal analysis. Guohua Zhang: resources, funding acquisition. Guofu Zhang: resources, funding acquisition. Miao Zhang: data curation and visualization. Shoufeng Xue: formal analysis and visualization. Huixiang Wang: writing–review & editing, conceptualization, methodology, formal analysis, and funding acquisition.

## Conflicts of interest

The authors declare no competing financial interest.

## Data Availability

The authors confirm that the data supporting the findings of this study are available within the article, and further inquiries can be directed to the corresponding author.
